# The Multi-Causal Basis of Developmental Potential Construction

**DOI:** 10.1007/s10441-023-09456-8

**Published:** 2023-01-30

**Authors:** Davide Vecchi, Gil Santos

**Affiliations:** Centro de Filosofia das Ciências, Departamento de História e Filosofia das Ciências, Faculdade de Ciências, Universidade de Lisboa, 1749-016 Lisbon, Portugal

**Keywords:** Developmental potential, Developmental entrenchment, Epigenetic landscape, Preformationism, Phenotype construction, Developmental novelty

## Abstract

In this article we analyse the issue of what accounts for developmental potential, i.e., the possible phenotypes a developing organism can manifest during ontogeny. We shall argue in favour of two theses. First, although the developing organism is the unit of development, the complete causal basis for its potential to develop does neither lie entirely in itself as a whole nor in any specific part of itself (such as its genome). Thus, the extra-organismal environment must be counted as one of the three necessary, partial and complementary causal bases for development potential. Secondly, we shall defend a constructivist view of the developmental process. If the genome, the developing organism and the extra-organismal environment are to be counted as proper elements of the causal basis for an organism’s developmental potential, the latter is not a given. Rather, it is the result of an interaction-based construction, a process sometimes generating genuine developmental novelty. We will thus argue for an interactionist multi-causal basis view of developmental potential construction. We contend that our view provides a biologically tenable and metaphysically coherent account of developmental dynamics.

## Introduction

Organisms are the units of development, since they are the entities acquiring particular phenotypes.^1^ But what makes them capable of developing? From where does their potential to develop come from? If such potential comes from the organisms themselves, do they get it from one of their particular components, such as the genome, or from the organisms’ global structure taken as a whole? And what is the causal role of the extra-organismal environment? To ask these questions is equivalent to ask *what grounds* or *accounts for* what might be called *developmental potential* (Waddington 1956 p. 351; West-Eberhard 2003 p. 13).

According to the contemporary philosophical literature on the topic of potentialities or dispositions (also called powers or capacities), potentials are properties that have their *manifestation* necessarily dependent on conditions other than the ones allowing their *instantiation*. This means that, unlike categorical properties, dispositional properties can be instantiated without being manifested. Specifically, they are properties which, if instantiated, only manifest themselves under certain external contingent conditions, often called ‘stimuli’, ‘manifestation’ or ‘triggering’ conditions (e.g., Hüttemann & Kaiser 2018, p. 402). Furthermore, according to the same literature, to enquire about what grounds or accounts for any disposition, such as developmental potential, is equivalent to ask about its *causal basis*. The causal basis of any potential, such as to be soluble, to be reproducible or to be capable of developing, is traditionally conceived as constituted by an actual structural property or set of structural properties, under the widely assumed principle that there are no ‘free-floating’ dispositions or potentials (with the only possible exception of those belonging to the ultimate level of physical reality). As Kistler and Gnassounou (2007) note, the theory called ‘dispositionalism’ does not deny, by definition, that a dispositional property, such as ‘being soluble’, must be grounded in the structure of the soluble substance. Dispositionalism “only rejects the metaphysical thesis that those grounding properties are necessarily categorical” (2007, p. 28). This means that one may well acknowledge the existence of both dispositional and categorical properties without necessarily endorsing the metaphysical view that goes by the name ‘dispositionalism’.

The aim of this article is not to argue for or against dispositionalism. Rather, we shall argue for a particular view about one specific biological dispositional property, i.e., developmental potential.

First, we shall argue that, although the organism is the unit of development, the potential for that development does not lie entirely in itself – either in a specific part of itself (such as its genome) or in itself taken as a whole. The extra-organismal environment must be counted as one of the causal bases for development potential. In this sense, we will argue that, even though developmental potential is a potential instantiated and only manifestable by individual organisms, it has a *multi-causal* basis, in the sense that its monadic instantiation often depends on potential sources that *go beyond* the boundaries of each organism. In this sense, developmental potential is an *extrinsic relational* property.^2^ This means that the causal basis of developmental potential cannot be accounted for from an exclusively *intrinsicness* viewpoint, for it might not be grounded exclusively on the intrinsic potentialities of the genomic and extra-genomic endogenous structures of an organism. As we shall argue, the developmental potential of every organism is causally grounded in a particular *relation* between (i) the potentialities of its genome, (ii) the potentialities of its extra-genomic endogenous structures, and (iii) the *extra-organismic* potentialities of certain environmental structures. Thus, the disposition ‘to develop’ has often a multi-causal basis with a combined *endo-exogenous* nature. In other words, the developmental potential that every organism possesses is *partially* grounded in the endogenous structure of the organism and *partially* grounded in the structure of the external environment.^3^

The second issue treated in the context of the present article concerns the best way to conceptualise the notion of developmental potential itself. In particular, we shall argue that, even though the genome, as one of the causal bases of developmental potential, may be seen as roughly structurally invariant on the timescale of ontogeny (but see note 5), the same cannot be said about the *non-genomic* intra-organismic and extra-organismic structures. Therefore, the full set of causal potentialities grounding the disposition of an organism to develop cannot be conceived from a *preformationist* viewpoint. As the intra and extra-organismic non-genomic structures undergo structural changes, their own sets of potentialities also change. Henceforth, the actual development of an organism cannot be conceived and explained as a mere activation of a particular subset of absolutely prefixed causal potentialities given ab initio. In addition to the presumably pre-fixed genomic potentials of an organism, we need also to take into account the *variable* potentialities of the non-genomic intra-organismic and extra-organismic changeable structures. Hence, against an essentially intrinsicalist and preformationist standpoint, we shall claim for an interaction-based *constructivist view* of developmental potential.

The position defended in this article is founded on important precedents. While developmental systems theory is naturally a general reference, the specific kind of developmental constructionism hereby endorsed is more properly based on the seminal biological work of Gilbert and Epel (2009) and, especially, West-Eberhard (2003), which permeates our philosophical analysis. Philosophically, our analysis is related to Love (2003) and, especially, McKitrick (2014).^4^ Our article makes a contribution to two general debates, the first concerning the causes of development (specifically the role of processes of assimilation and functional integration of environmental resources at the origin of developmental novelty) and the second regarding the nature of a developmental system’s potential within the conceptual framework provided by current debates on the nature of dispositional properties in biology (specifically the intrinsic vs. extrinsic issue). More specifically, the elements of novelty of our analysis concern its robust biological foundation (replete with realistic illustrative examples), the argument in favour of the endo-exogenous basis of developmental potential and, finally, the proposal of a relational view of development.

Our analysis has the following structure. In Sect. 2 we shall clarify what structures are involved in developmental processes and categorise the different views concerning the causal basis of developmental potential. In Sect. 3 and 4 we shall analyse and criticise preformationist genomic potentialism. In Sect. 5 we shall analyse and criticise a more encompassing view, focusing on the developing organism as the unit to which developmental potential is ascribed. In Sect. 6 we shall articulate what we consider the only biologically tenable and metaphysically coherent characterisation of developmental potential.

## The Triad of Developmental Structures and the Four Possible ‘causal bases’ of Developmental Potential

Development or ‘phenogenesis’ (Sarkar 2005) can be characterised in many ways, some restrictive and some less so. We favour the latter avenue and characterise development, following West-Eberhard (2003, pp. 89–90) and Mahner & Bunge (1997, pp. 271–6), as the series of qualitative changes a responsive biological system undergoes during ontogeny due to genomic and extra-genomic causal influence. By qualitative changes we refer to the changes in the composition, organisation or function of the responsive biological system. Thus, any molecular process (e.g., DNA replication, transcription and translation) is here considered a developmental process. If development is characterised in these general terms, every organism, by undergoing qualitative changes during its life history, develops.

In order to characterise developmental potential, it is conceptually useful to discriminate between three types of structures involved in developmental processes: the genome, the developing organism and the environment. We shall assume that the genome is a system that remains largely organisationally stable through development and that is largely structurally identical in all cells of the developing organism (apart from somatic mutations).^5^ The developing organism is a system that continuously changes compositionally, organisationally and functionally during ontogeny. The developing organism grows, undergoes morphogenetic changes and its parts differentiate. Some of these changes are caused by the assimilation, functional integration and eventual deployment of environmental resources, a process that West-Eberhard (2003, pp. 500 ff.) calls “developmental entrenchment”. These resources can be categorised as either environmental *inputs* (e.g., a specific temperature affecting cellular interactions during sexual differentiation) or as environmental *materials*, i.e., building blocks in phenotype production (e.g., assimilated amino acids deployed in protein synthesis).^6^ Despite the continuous compositional, organisational and functional changes the developing organism undergoes during ontogeny, we assume that one form of relative stability is given by the genome. The environment can be conceptualised as the set of developmental resources available to a system of reference: if the system of reference is the genome, the relevant environment is extra-genomic, including all the set of molecular resources to process DNA and RNA molecules in replication and transcription; if the system of reference is the developing organism, the relevant environment is the external environment to the organism itself. Importantly, given that the developing organism is a continuously changing biological system, the external environment can only be characterised vis-à-vis a particular developmental stage rather than generally. For instance, the external environment of a developing foetus includes the uterus, while after delivery it is extra-uterine. Equally important, the environment provides a constantly changing set of developmental resources to the developing organism, as it constantly undergoes modifications caused abiotically and biotically.

This characterisation of development allows us to distinguish mere qualitative developmental changes from developmental novelties. Only qualitative developmental changes that never occurred in the history of lineage to which the developing organism belongs are developmental novelties.^7^ Evolutionary novelty requires the heritability (and increase in frequency in the lineage) of developmental novelty. In this sense, developmental novelties are the essence of the evolutionary process (Mahner and Bunge 1997, pp. 313–316).

Given this triad of structures, we can identify four possible characterisations of developmental potential. Our chief aim is to distinguish logical positions independently of their actual, especially contemporary, endorsement. This characterisation will serve the important analytic purpose of uncovering their biological and metaphysical foundations. The first three characterisations correspond to a mono-causal basis view, either centring on the genome, the organism or the environment. The first grounds the notion of developmental potential merely in terms of the causal capacities of genomes. This view can be labelled *preformationist genomic potentialism* and is based on the postulation of some form of genomically fixed and pre-set developmental potential. This view is preformationist and intrinsicalist in nature. In the first sense, the organismal genome contains in a preformed state all the possible phenotypes the organism can eventually develop during ontogeny. In the second sense, it is only the organismal genome that possesses from the outset the generative capacities that cause all possible phenotypic effects during development. Development is, according to this view, just the selective manifestation of some of the preformed and intrinsic potentials of the genome, which lie in a dormant and latent state, waiting to be triggered appropriately by the environment. Thus, the relevant environment plays a causal role in development, but just as an appropriate selective triggering cause in producing a particular dispositional effect in the form of a specific phenotypic outcome. Needless to say, this view has a long tradition in developmental biology and philosophy of biology (Weismann 1893; Wolpert and Lewis 1975; Rosenberg 1997; Austin 2015 among many others).

One alternative is to view the whole organism (including, of course, its genome) as the causal basis of developmental potential. The historical roots of this view are also old. The basic idea is internalist and organicist in ethos (St. Hilaire 1818, Maturana & Varela 1991, Newman 2021, Austin and Nuño de la Rosa 2021 among many others): developmental potential is grounded in the causal capacities of developing organisms, specifically in their material constitution and structural organisation. Even though the focus of this view is not merely on one component of developing organisms (i.e., the genome), clearly taking into consideration those extra-genomic factors that are internal to the developing organism, the entire developing organism becomes the only relevant causal basis of developmental potential. Again, if the environment is merely seen as an appropriate selective triggering cause in producing a particular dispositional effect in the form of a specific phenotypic outcome, this view remains preformationist and intrinsicalist, thus becoming a more encompassing form of preformationism that could be labelled *preformationist organismal potentialism*. In other words, within these two views, the causal basis of any organism’s developmental potential lies entirely in the organism itself*.* Ultimately, the environment just acts by *triggering* the manifestation of the potentialities of either the genome or of the developing organism, thus only providing the appropriate ‘background manifestation conditions’*.*

The third possible mono-causal basis view of developmental potential might be labelled *environmental potentialism*. This view is epigenetic and externalist in nature. In the first sense, it denies that the developing organism (including its genome), at any particular developmental stage, contains in a preformed state all the possible phenotypes the organism can eventually develop during ontogeny. In the second sense, it denies that the developing organism (including its genome), at any particular developmental stage, possesses the intrinsic generative capacities that cause all possible phenotypic effects during development. Development is, according to this view, to be accounted for merely in terms of environmental contribution. Thus, the relevant environment always plays a constructive role in development rather than being merely a triggering cause in producing specific phenotypic outcomes. This would make the developing organism (including its genome) causally irrelevant structures in development because, independently of their nature, the same phenotypic outcome might result. The causal influence of the developing organism (including its genome) would be always overridden by the causal influence of the environment.

Let us now emphasise an asymmetry: while it is currently difficult to find advocates, amongst biologists and/or philosophers, of environmental potentialism (despite historical precedents, e.g., Watson 2009, p. 82), preformationist views have a long history of advocacy. We shall criticise these views in Sects. 3, 4 and 5, an endeavour that will require biological as well as metaphysical considerations. As we shall argue, any characterisation of developmental potential is implicitly grounded on a more or less hidden metaphysical outlook.

The fourth view concerning the nature of the causal basis of developmental potential is, of course, that each of the above-mentioned three types of structures participating in development is a partial and complementary causal basis of developmental potential. The historical roots of this position are probably more recent (West-Eberhard 2003; Gilbert and Epel 2009) and the chief aim of this article is to contribute to its articulation and defence. This *multi-causal basis view*, a full characterisation of which will be provided in Sect. 6, can be presented as an *interactionist multi-causal basis view of developmental potential construction*.

## Preformationist Genomic Potentialism

The concept of genomic potential has an intuitive biological basis: after all, bacteria beget bacteria and humans beget humans. The observable unity of type (i.e., the phenotypic similarities between the organisms of the same taxon) is surely partially dependent on the genome. Of course, that the genome plays a causal role in development is not the interesting tenet of preformationist genomic potentialism. In order to make sense of the concept of genomic potential it is necessary to uphold three additional theses:

- The genome limits developmental plasticity;
- Development is a conservative process;
- Development is conceptualised in predeterministic terms.

Let us now consider the underpinnings of these theses in turn.

### Limits to Developmental Plasticity

**Fig. 1:**
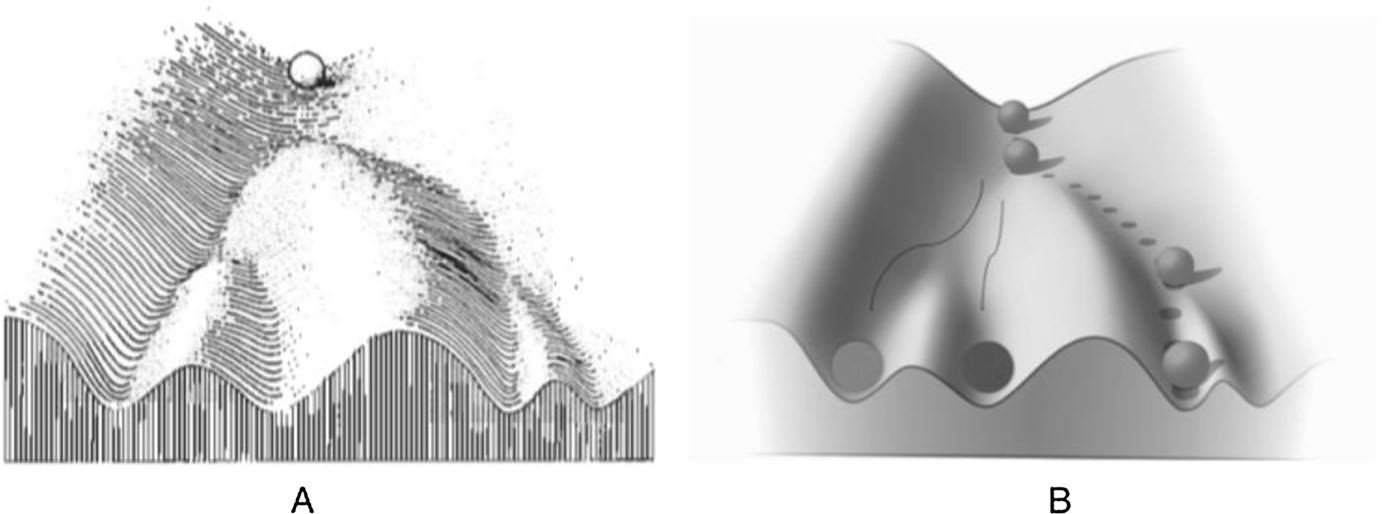
**A** Waddington’s representation of the epigenetic landscape. **B** Epigenetic landscape representing the three viable phenotypes (i.e., the differently circled balls at the bottom of the slope) that can be manifested by the developing organism (i.e., the ball at the top of the slope)

**Fig. 2:**
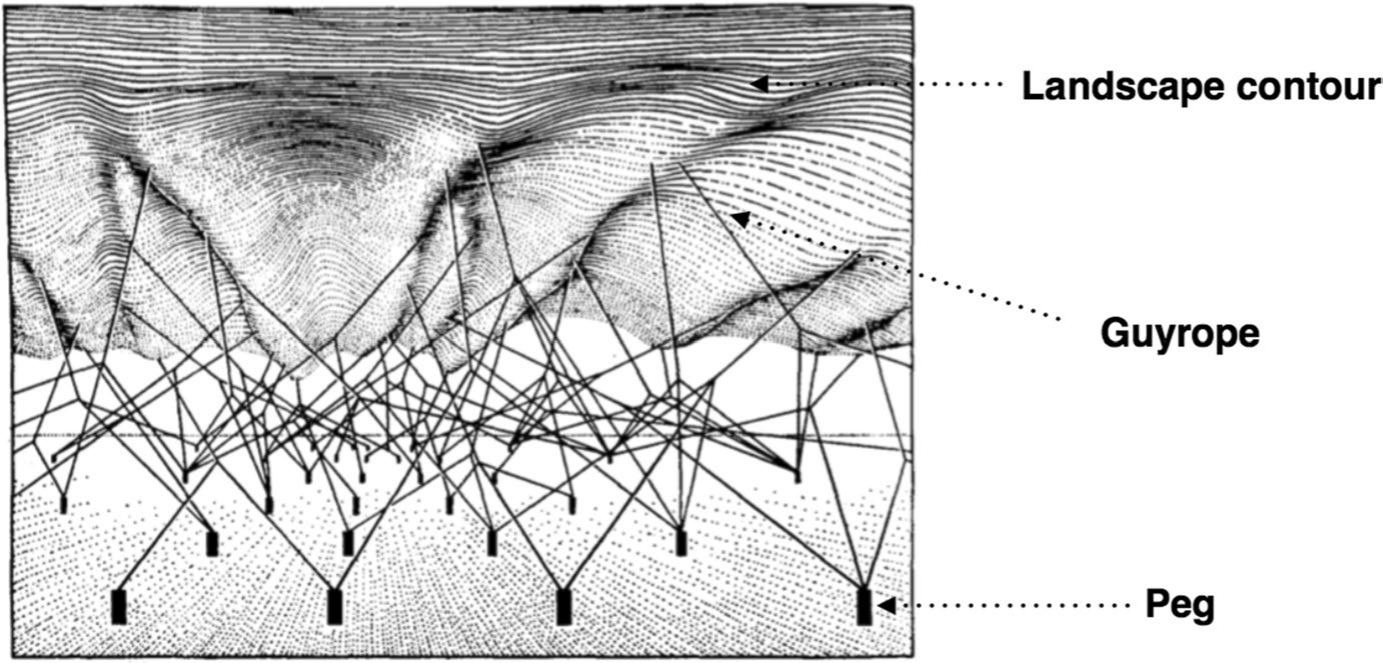
The underlying structure of the epigenetic landscape with pegs as genes and guyropes as genes’ “chemical tendencies” determining the topology of the contour

The way in which the genome limits plasticity is captured by the concept of *reaction range*. Developmental psychologists have coined this concept in order to represent the limits of the reaction norm (Anderson Platt & Sanislow 1988). The reaction norm is specific to a given genotype. The idea of reaction range is that, given a genotype, the set of phenotypes the developing organism can manifest has a fixed range, with an upper and lower limit determined by genomic resources. Thus, no possible environment can expand it. Despite its reference to a specific genotype, the concept can be generalised to apply to the entire genome.^8^ This slippage from the *genotype concept of reaction* range to the *genome concept of reaction range* appears frequently in the psychological literature. For instance, it is unclear which reaction range concept is used by Atkinson et al. (1987, p. 409): “…genes do not specify behavior; rather, they establish a range of probable responses to the environment, which is called the *reaction range.*” The underlying idea to the genome concept of reaction range is that the genome of the individual organism sets the limits, while the environment contributes, within those limits, to the specificity of the phenotype. One way in which the genome sets limits to developmental plasticity is by closing the reaction range, determining its lower and upper boundaries. It has been argued (Wahlsten & Gottlieb 1997, p. 172) that the concept of reaction range comes naturally from Waddington’s conceptualisation of development. There might be some truth in this interpretation (Sarkar 2005, chapter 14; West-Eberhard 2003, pp. 13–16; Gilbert 1991). Consider Waddington’s (1957, Fig. 4, p. 29) famous representation of the epigenetic landscape (Fig. 1A):

Waddington (1956, p. 351, caption to Fig. 16.2) considered the epigenetic landscape “a symbolic representation of the developmental potentialities of a genotype”. The landscape contour has a particular topology: the developing organism (the ball at the top of the slope) may develop in several ways depending on which side of the slopes it takes. In Fig. 1B a series of possible viable phenotypic outcomes is represented. The graphical representation gives the impression that there are only three viable developmental outcomes. Of course, this is a simplification. Instead of a very small number of developmental outcomes, it is better to think about an indefinite but circumscribed set (see Anderson Platt et al. 1988, p. 255). The developmental outcomes at the far left and far right of the landscape represent the limits of the fixed and closed genome reaction range of the individual organism. Given this conceptualisation, we can ask what causal factors can change the contour of the landscape. In order to answer this question, take a look at Fig. 2, depicting the underlying structure of the landscape (an elaboration of Fig. 5 in Waddington 1957, p. 36):

The pegs represent genes and the guyropes their “chemical tendencies”. The pegs can be analogised to the “geological structure” underlying the landscape contour (Gilbert 1991, p. 148). This representation conveys the idea that the only way to change the contour of the landscape is either by changing the pegs or the direction/pull of the guyropes. The changing of pegs occurs by genomic change (e.g., point mutation). Crucially, *if changing the direction/pull of the guyropes is only possible through genomic change, then the contour of the landscape is genomically determined* (see Sect. 4.2.2 for an analysis of this thesis). As a consequence, in the absence of genomic change, the contour would be static. This interpretation also conveys the thesis that developmental potentialities are predetermined by the genome.

According to preformationist genomic potentialism, the reaction range of the developing organism is closed, so that phenotypic manifestation has upper and lower limits. Furthermore, development is canalised along an indefinite but circumscribed set of routes, ranging between the upper and lower limits of the reaction range. When every route is conceptualised as ultimately dependent on the genome, phenotypic variability is somehow ‘encoded’ by the genome.

### Development is a Conservative Process

If the genome has a constraining power exerted by limiting and fixing the set of possible phenotypes at the outset, what is the role of the extra-genomic environment?^9^ We shall now take a look at two ways to understand environmental influence in development that can be grasped by considering again Waddington’s representations of the epigenetic landscape.

**Fig. 3:**
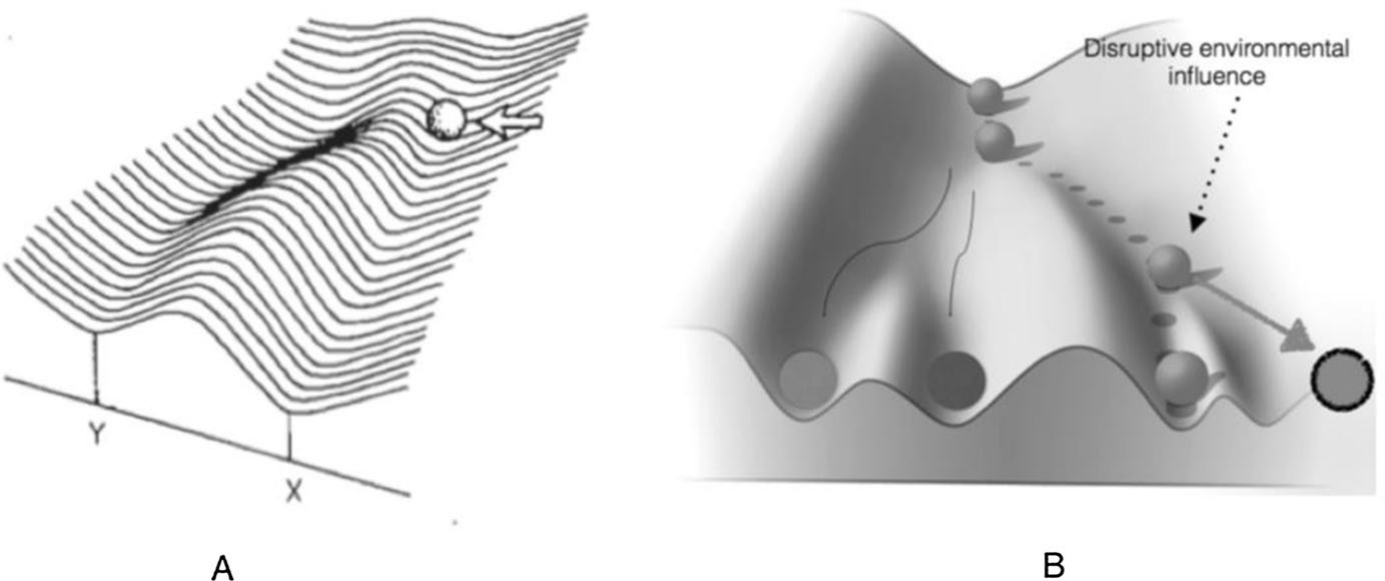
**A** External environmental influence (i.e., the white arrow) impinges on the developing organism (i.e., the ball) by deflecting its developmental pathway (leading to the manifestation of phenotype Y). X and Y are the viable phenotypes that can be manifested by the developing organism. **B** Disruptive environmental influence ‘triggering’ the manifestation of an unviable phenotype (i.e., the circled ball on the right)

One first sense in which the environment can influence development is at the heart of the idea of canalisation along an indefinite but circumscribed set of developmental possibilities. Consider Fig. 3A, the graphical representation of this kind of environmental influence proposed by Waddington (1957, Fig. 30 top, p. 167):

The idea conveyed by the representation is that external environmental influence (i.e., the white arrow) impinges on the developing organism (i.e., the ball) by deflecting its developmental pathway along a different slope of the landscape contour. But the phenotypic outcome (i.e., Y or X or, in Fig. 1B, the three viable outcomes) is by definition determined by the genome in case the contour of the landscape is genomically determined in the sense specified in Sect. 3.1. In this case environmental influence basically triggers the manifestation of a developmental potentiality or intrinsic disposition of the developing organism set from the outset by the genome. For instance, a specific temperature will cause, *ceteris paribus* (given the same genomic and organismal profile), a specific sex: either male (e.g., X) or female (e.g., Y). So, all the possible phenotypic outcomes are merely causally triggered by the environment but pre-set by the genome.

A second possibility is that environmental influence is disruptive (Fig. 3B), that is, that it generates a phenotype that, first, is not part of the indefinite but circumscribed array of pre-set developmental possibilities allowed by the genome (i.e., the circled ball on the right) and, secondly, that is unviable.

A disruptive environmentally-induced event of this kind might have various kinds of effects. For instance, a low temperature might lead to the death of the developing organism. Or the environment (e.g., X-ray radiation) might induce deleterious genomic changes (i.e., changes in the pegs) leading to the death of the developing organism. In the latter case, disruptive environmental influence is mediated by genomic change, producing either somatic or germ-line mutations. Developmentally speaking, mutations might either be neutral (whereby the change in pegs does not change the contour), advantageous (whereby the change in pegs changes the contour, producing qualitative developmental changes with novelty potential) or deleterious (whereby the change in pegs indeed changes the contour but produces disruptive qualitative developmental changes with no possible novelty potential). If it is assumed that most non-neutral developmentally relevant mutations are deleterious, then environmental influence is mostly disruptive. The few advantageous mutations remaining will provide the raw material for evolutionary novelty.

The upshot of this view is that the causal role of the environment is limited to either triggering the manifestation of an already existent developmental potentiality established by the genome or to some form of disruption and developmentally deleterious effect. Thus, environmental influence is either canalised or disruptive at most, but certainly not creative in the sense of generating novel phenotypes not fixed from the outset by the genome. Furthermore, when the environment causes developmentally advantageous genomic changes, it is the genome that more proximally causes novelty. In brief, development is conceptualised as a generally conservative process (West-Eberhard 2003, p. 13 ff), while the origins of novelty are reduced to genomic change. Thus, the environment does not participate actively in the *construction* of the phenotype.

### The Predeterministic Conceptualisation of Development

Development becomes, according to this view, a process of manifestation of the intrinsic potential of the genome, a potential that lies in a preformed, dormant and latent state, waiting to be environmentally triggered appropriately. In this sense, the genome determines (in a preformationist fashion) all properties that the developing organism may manifest in the future (i.e., all the phenotypes it may manifest during ontogeny).

Arguments defending the ontological distinctiveness of DNA (for a critical review see Vecchi 2020) provide the ideal support for this metaphysical view. For instance, Austin (2015) distinguishes two kinds of causal contribution in developmental processes: causal ‘relevance’ and causal ‘responsibility’. Austin argues that only the genome is causally ‘responsible’ in development.^10^ The reason can be grasped by considering the structure of the epigenetic landscape: it is only the genome that determines the specific nature of the contour of the landscape and that, consequently, is ‘responsible’ for the existence of its various canals with those specific topological features.

The difference between competence and induction articulated by Waddington (Gilbert 1991) further substantiates this predeterministic conceptualisation of development. Waddington thought that the *competence* of the cells of the developing organism determined the phenotypic outcomes generated by developmental processes. Given that competence is genetically controlled, the thesis that the genome is causally ‘responsible’ for phenotypic outcomes ensues. The graphical representation (Fig. 1) of the nature of developmental pathways makes visible this sense of causal responsibility: as soon as the cell enters the pathway, its fate becomes increasingly fixed the higher the pathway’s canalisation, which is determined by the genetic “geological structure”. Competence is thus preformed because it exists in the competent cell prior to *induction*. However, the extra-genomic environment plays a role in development. Any qualitative developmental change is in fact *induced* by a variety of natural or artificial compounds. Inducers can also be external or internal (vis-à-vis the cell) factors. The external factor friction induces callus formation in humans by triggering the genetically determined and preformed causal capacities of competent skin cells. The preformed competence of skin cells could eventually be induced by an internal factor in the absence of the external factor. So, in ostriches, a mutation allowed skin cells to respond to an inducing compound internal to the developing organism, leading to what Waddington (1957, Fig. 30 p. 167, lower diagrams) called “genetic assimilation” (Gilbert 1991 pp. 148–9). This way of conceptualising developmental processes thus distinguishes induction and competence: while the first process is due to inducers conceptualised as triggering factors that push cells towards specific canalised pathways, competence is genomically determined and preformed.

## Debunking Preformationist Genomic Potentialism

In this section we shall identify the limitations of preformationist genomic potentialism. We shall argue that the causal role of the extra-genomic environment is often not clearly under the control of the genome and that, therefore, it can be seen as an autonomous source of new developmental potentialities.

### The Reaction Range is Not Fixed

The first significant feature of preformationist genomic potentialism concerns the existence of a reaction range. The genotype concept of reaction range has been previously criticised:

“The norm of reaction of a genotype is at best only incompletely known. Complete knowledge of a norm of reaction would require placing the carriers of a given genotype in all possible environments, and observing the phenotypes that develop. This is a practical impossibility. The existing variety of environments is immense, and new environments are constantly produced. Invention of a new drug, a new diet, a new type of housing, a new educational system, a new political regime introduces new environments.” (Dobzhansky 1955, pp. 74–75).

Dobzhansky makes both an epistemological and an ontological point. The former concerns the impossibility of knowing the putative upper and lower boundaries of the reaction norm. Along the same lines, Anderson Platt et al. (1998, p. 256) argue that the genotype’s reaction range is a theoretical imposition with no empirical justification because its upper and lower boundaries “are not really reflective of a genotype per se” but, rather, of our ignorance. Dobzhansky’s ontological point highlights the fundamental problem that, given that new environments are constantly produced, the idea of a closed reaction norm is incoherent. Along the same lines, Sober (1980, p. 376) suggests that it is difficult to make sense of the idea that the reaction norm should be thought abstractly as also encompassing the causal influence of those environments not yet encountered by the organisms of the lineage or even not yet existing in any meaningful biological sense. The fundamental point is that there is no good biological reason to assume that the genotype fixes the norm of reaction in the absence of evidence concerning the possibility that its expansion is due to extra-genomic environmental influence. In this sense, the genotypic reaction range concept becomes incoherent in case its boundaries are extended whenever the organism displays a phenotype outside the range (as it is supposed to be fixed, with an upper and lower limit determined by genomic resources, see Sect. 3.1). Given that the genome reaction range concept is a generalisation of the genotype concept, this should be sufficient to show that it is problematic. But the problem goes deeper and is symptomatic of the metaphysical, supra-empirical character of preformationist genomic potentialism. To articulate this point, let us now consider two examples.

In Sect. 2 we pointed out that the environment provides a constantly changing set of developmental resources to the developing organism as it constantly undergoes modifications caused abiotically and biotically. Consider two environmental materials not directly biosynthesized by the developing organism. For instance, silicon is an omnipresent and abundant chemical element in the environment of plants. Thus, the presence of silicon does not make the environment novel in any sense.^11^ Silicon was not generally considered a physiologically ‘essential’ material for plants’ development, i.e., a necessary developmental resource to complete the plant’s life cycle (Epstein 1994). Nonetheless, some plants, for instance rice, have managed to functionally integrate silicon and deploy it for a specific function in cell wall formation, thus generating a novel phenotype: resistance to fungal infection (Wang et al. 2017). That is, an abiotic environmental material has been assimilated, functionally integrated and ultimately deployed as a developmental resource in a novel way by rice. The same can be said about biotic environmental materials. For instance, the alkaloid batrachotoxin is assimilated by the golden poison frog *Phyllobates terribilis* by eating batrachotoxin-containing insects (Dumbacher et al. 2004). Here the environment has an element of novelty because batrachotoxins need to emerge in the lineage of batrachotoxin-containing insects in the first place and such insects must become part of the frogs’ environment. In this case, the assimilation process was most probably accompanied by a reduction in population size (batrachotoxins are, after all, toxins). When batrachotoxins are functionally integrated and deployed for protection from predators, a biotic environmental material becomes a developmental resource in this frog lineage. These two cases of “developmental entrenchment” are arguably significant evolutionary novelties in the history of the respective lineages (West-Eberhard 2003, p. 502–3). Because of environmental contribution, development is not a conservative, but a creative process (see Sect. 3.2). We would argue that, in both cases, the developmental entrenchment of the environmental material for a specific function has opened the supposedly fixed genome reaction range of the ancestral organism of the lineage that first developed the novel phenotype. If we are right, the genome concept of reaction range is problematic.

Let us now consider two counterarguments. The first states that the above cases of developmental entrenchment cannot occur without preceding genomic changes. We would argue that this argument is based on two unsubstantiated assumptions. The first is that there must be a different genomic constitution between the ancestral and contemporary organisms of the lineage concerning the specific genomic capacities associated with the emergence of the developmental novelty. The second is that the different genomic capacities were acquired by the gradual accumulation of genomic changes. Needless to say, the above examples involve a multi-generational dynamic.^12^ In this sense, we do not dispute that genomic change has occurred in the frog and rice lineages. What we dispute is rather that genomic change has necessarily affected the genomic capacities associated with the emergence of the developmental novelty. Whether this actually happened is an open empirical question. To assume otherwise is an expression of a geno-centric bias tailored to a priori dismiss the causal role of the extra-genomic environment in development. Put differently, the point we would like to stress is that the extra-genomic environment might generate developmental novelty in the absence of genomic change, as hypothesized by the ‘phenotype-first’ scenario of evolutionary novelty. Thus, the assimilation and functional integration of ubiquitous environmental materials should not be assumed to occur because of previously accumulated genomic change.^13^

The second counterargument shows, in our opinion, that preformationist genomic potentialism is a Janus-faced position. The argument can be put as follows: even supposing that the developing organism developmentally entrenches the new material and generates a novel phenotype, the preformationist genomic potentialist would write off this causal contribution by arguing that the environmental material merely ‘triggers’ the genomically determined and preformed developmental potential of the organism. Any kind of developmental novelty would ultimately be attributed to the genome. In the case of both reaction range concepts, any extension of the range would be attributed to the underlying genomic capacities of the developing organism. Suppose two genomically identical developing organisms live in two different environments. Suppose the difference just concerns the absence or presence of a new environmental material. Is it possible to think that only the organism developing in the new environment manifests a novel phenotype outside of the reaction range? If the preformationist genomic potentialist dismisses this possibility or, alternatively, argues that this would only be possible because of the unexpressed, dormant but still preformed and genomically determined capacities of the organism, then preformationist genomic potentialism will be more clearly seen for what it actually is, a metaphysical position disguised as an empirical hypothesis. The first reply seems spurious, while the second is based on the sophistic point that the novel phenotype can be manifested not because the environmental material has been developmentally entrenched, but because developmental entrenchment has been made possible by the genome. It is this asymmetry in considering the intrinsic and extrinsic causal bases of developmental potential that seems to us unjustifiable. The only way to criticize it is by appealing to more general theoretical and metaphysical considerations, as we shall do in Sect. 6.^14^

Ultimately, we would argue that both concepts of reaction range are nebulous. There is no good empirical or theoretical reason to doubt that the extra-genomic environment (i.e., the variety of developmental resources that either developing organisms possess intrinsically or extrinsically) can generate developmental novelty. As we shall argue in Sect. 4.2, there are indeed good theoretical reasons to think otherwise and to reject the geno-centric view dismissing the causal role of the extra-genomic environment in development.

### Genomic ‘Encoding’ is a Metaphor

**Fig. 4:**
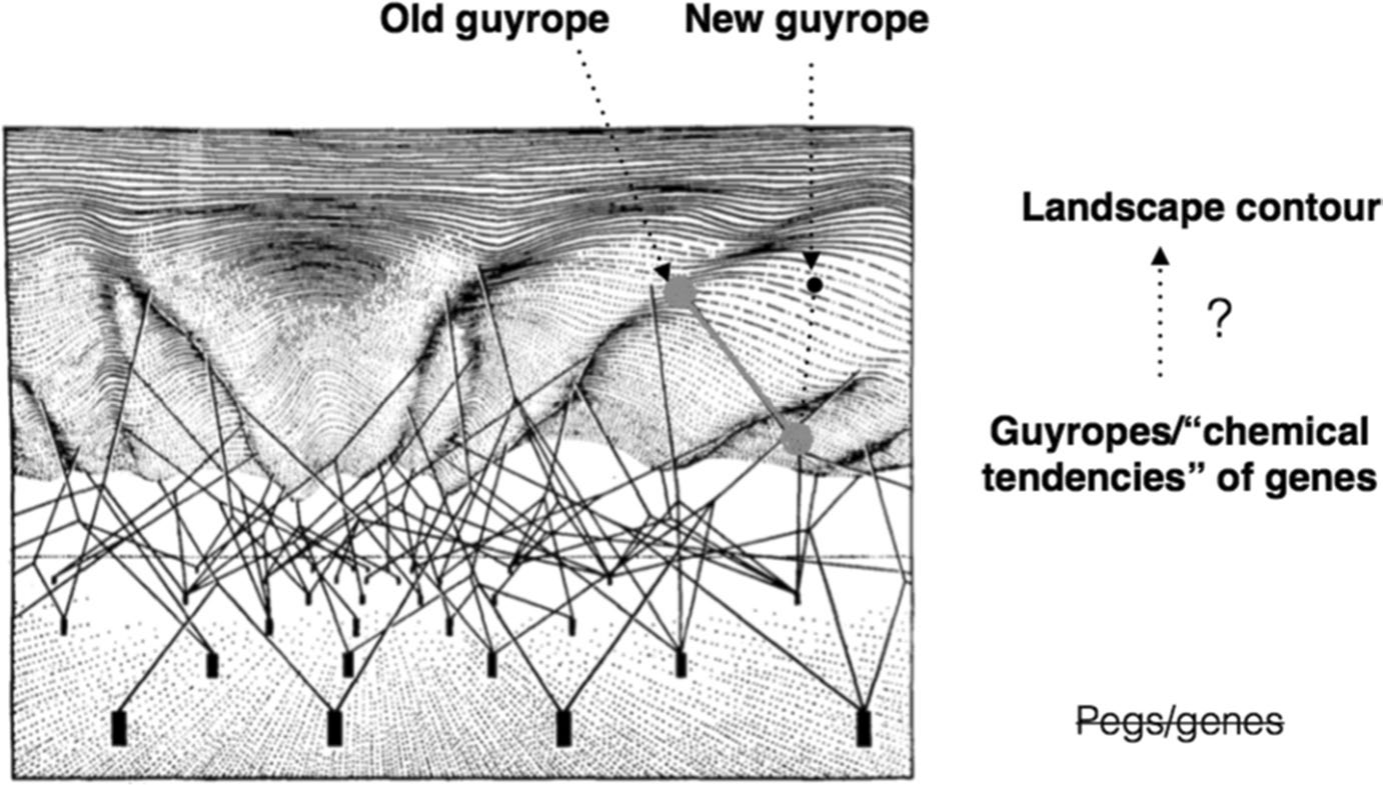
Extra-genomic causal factors re-direct the guyrope (from old to new): might this re-direction engender a change in the epigenetic landscape contour without genomic change?

As we saw in Sect. 3.2, if development is canalised along an indefinite but circumscribed set of routes ranging between the upper and lower limits of the reaction range and, moreover, if the topological features of the routes are ultimately dependent on the genome, phenotypic variability might be said to be somehow ‘encoded’ by the genome. There is no doubt that some phenotypes are strongly dependent on the genome. For instance, it is said that proteins are ‘coded’ by genes. This means that, *ceteris paribus*, from a gene a certain protein with a specific native structure will result. In this restricted *ceteris paribus* sense, the process of protein biosynthesis is considered insensitive to the vagaries of the extra-genomic environment. But the *ceteris paribus* clause refers to a ‘normal’ extra-genomic environment, i.e., an environment with the ‘right’ developmental resources. If the ‘right’ conditions are not in place, protein biosynthesis might be impaired unless the cell has the capacity to override the ensuing ‘errors’. This prelude is relevant because Waddington’s representation of the structure of the epigenetic landscape (see Fig. 2) makes possible to envisage the possibility (see Fig. 4) that *extra-genomic factors might causally affect the directionality or pull of the guyropes providing the scaffolding of the epigenetic landscape contour, thus engendering a change in the contour without genomic change (i.e., the change in the composition or structural organisation of the pegs).*

If this were possible, then the landscape contour would not be genomically determined and developmental potential would not be genomically encoded. Let us analyse this issue.

#### Is Extra-Genomic Causal Influence Without Genomic Change Merely Disruptive?

**Fig. 5:**
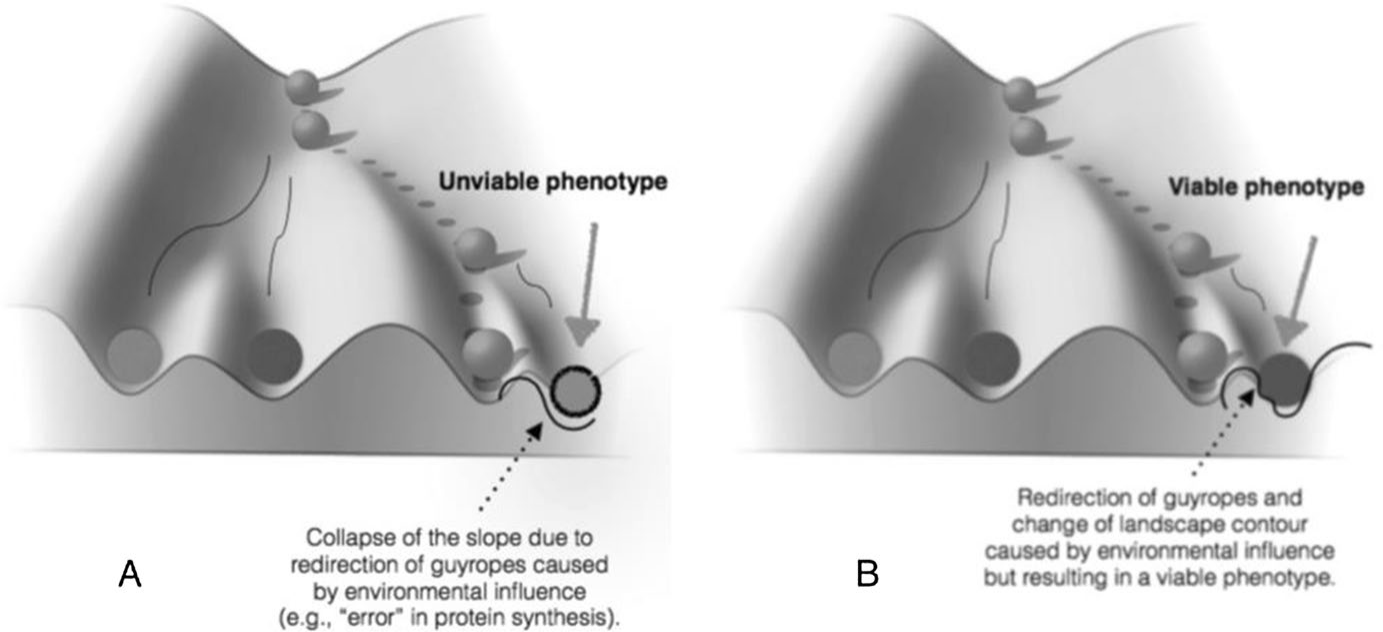
**A** Extra-genomic factors engender a change in the direction of the guyropes which in turn changes the epigenetic landscape contour, but the phenotypic outcome is unviable. **B** Extra-genomic factors engender a change in the direction of the guyropes which in turn changes the epigenetic landscape contour resulting in a viable phenotypic outcome

In Sect. 3.2 we clarified that, interpreted in terms of Waddington’s epigenetic landscape representation, preformationist genomic potentialism might consider extra-genomic causal influence in development as merely disruptive. Probably the simplest way to make sense of this hypothesis is to think about an error in protein synthesis. Implicit in the notion of error is the notion of damage, apparently making extra-genomic causal influence disruptive by definition. We can envisage that the change in the direction of the guyropes will change the contour as to render the phenotypic outcome unviable (Fig. 5A).

However, this generalised view concerning extra-genomic causal influence is untenable because some ‘errors’ in protein synthesis might produce functional phenotypes. At least in some cases, the most appropriate representation of the causal role of environmental influence is Fig. 5B. Let us explain what we mean by means of a variety of illustrative examples.

#### In What Sense Might The Landscape Contour Be Changed By Extra-Genomic Causal Factors in a Non-Disruptive Way?

**Fig. 6:**
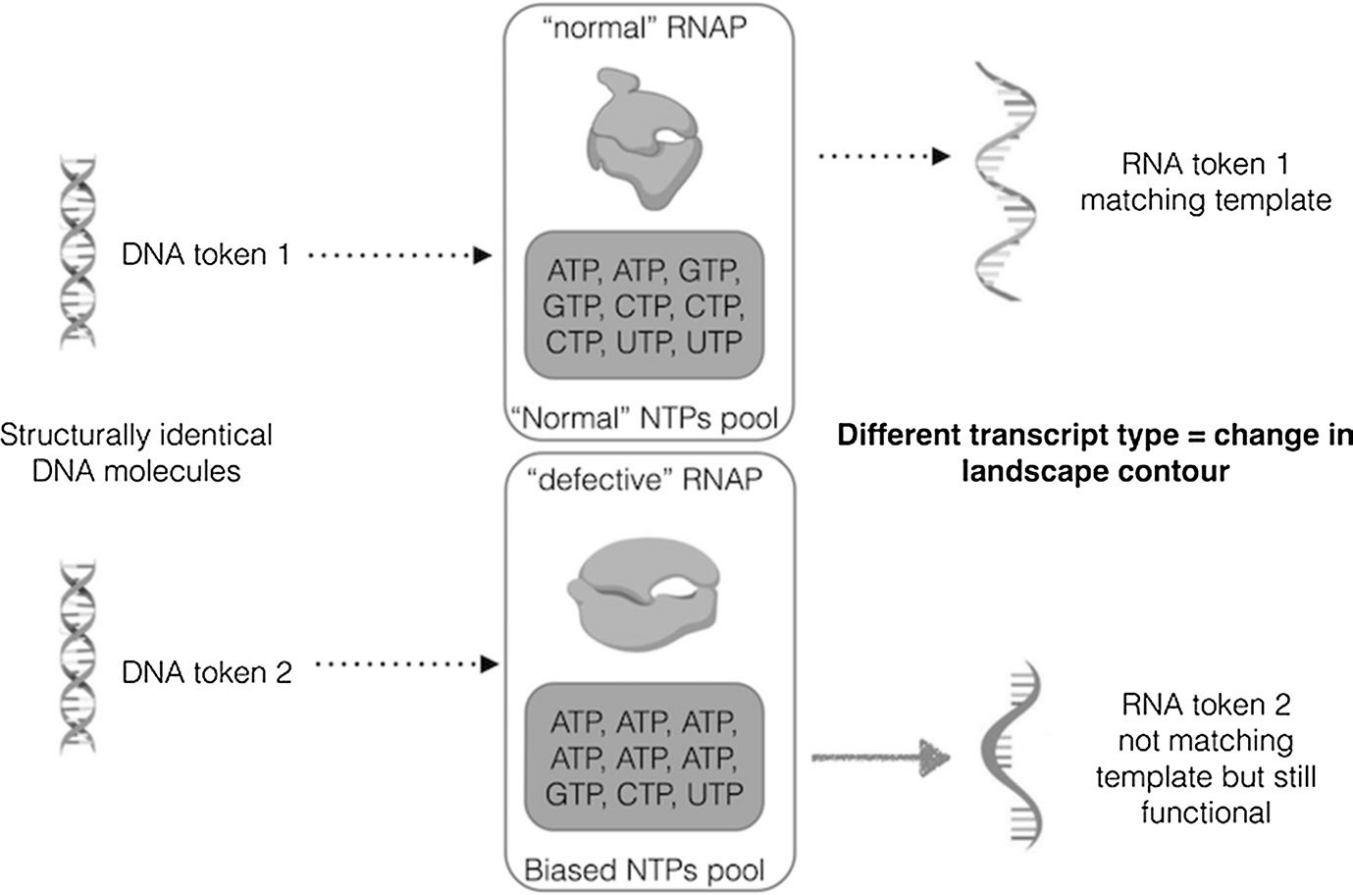
Two structurally identical DNA token molecules are transcribed differently. In the second scenario, this occurs either because the ‘defective’ RNAP is more error-prone (vis-à-vis the ‘normal’ RNAP) or because ATP is preponderant in the NTPs pool, illustrating that the genome is not causally ‘responsible’ for the ensuing difference. Thus, the second scenario shows how extra-genomic factors engender a change in the epigenetic landscape contour resulting in a viable phenotype (i.e., RNA token transcript 2), as represented in Fig. 8

The notion of error in protein synthesis is not normative: the ensuing phenotype is not necessarily lacking function and is not inevitably degraded by the developing organism. When transcription and translation are concerned, ‘error’ just means mismatch between nucleotide template (DNA or RNA-based) and phenotype (respectively, RNA transcript or polypeptide). Such errors occur frequently and for a variety of reasons that are only understandable if protein biosynthesis is not black-boxed. Arguments in support of the ontological distinctiveness of DNA are partially an artefact of such black boxing (Vecchi 2020). A transcription error might be, for instance, due to an error-prone RNA polymerase (henceforth RNAP) and/or to a biased distribution of nucleoside triphosphate (henceforth NTP) precursors in its vicinity (e.g., a higher concentration of ATP surrounding the RNAP will bias its performance by increasing the probability of mis-incorporating adenine in the transcript independently of the nature of the template). In both cases, (the same or) two structurally identical DNA token molecules might be transcribed differently, showing that the genome is not causally ‘responsible’ for the ensuing difference, i.e., for the occurrence of this kind of qualitative developmental changes (Fig. 6).^15^

In case the ‘erroneous’ transcript is used in protein synthesis, a different amino acid might end up being part of the polypeptide; in case this difference in amino acid composition changes the structure and function of the synthesised protein, what is the sense in saying that the ensuing phenotype (potentially a developmental novelty) is genomically ‘encoded’? This theoretical example in our opinion shows that extra-genomic causal factors (e.g., an error-prone RNAP or a biased distribution of NTPs) seem to be capable of changing the directionality or pull of the guyropes resulting in a change in the landscape contour without genomic change, engendering qualitative developmental changes that are in no sense encoded by the genome.

The same point can be analogously shown for any kind of post-transcriptional error. It is sometimes assumed that the primary structure of proteins determines its structure and function (Santos et al. 2020). The primary structure, in its turn, is assumed to be determined by the genome (as it is, *ex hypothesi*, ‘encoded’ by it). The underlying hypothesis is that only genomic change can alter primary structure and henceforth the structure and function of folded proteins: only change in the pegs will change the contour of the landscape and, henceforth, phenotypic manifestation. But this view is biologically incorrect for parallel reasons to those detailed above concerning the relationship between DNA and RNA molecules. Bifunctional proteins, for instance, possess same primary structure developing into the same native structure but performing two different functions. How do we explain phenotypic variability in the light of genomic, amino-acidic and structural sameness? One possible explanation is to identify an extra-genomic difference maker that might be conceptualised as changing the directionality or pull of the guyropes and, henceforth, landscape contour. An explanation of this kind might stress that, given that proteins often possess multiple active sites, the contingent presence of particular substrates (e.g., glucose or fructose) in the relevant environment (i.e., cellular or extra-cellular), i.e., an extra-genomic causal factor, is the difference maker causing the occurrence of one of the two phenotypes. If we assume that one of the two functions of the protein in a lineage is ancestral, an extra-genomic causal factor might be thought to be at the origin of a developmental novelty generating a new function, potentially creating evolutionary novelty.^16^ The contingent presence of specific extra-genomic developmental resources can thus affect development and originate potentially novel molecular phenotypes in the absence of genomic change. Probably the clearest example of this kind of dynamic concerns the causal role of prosthetic groups (e.g., chlorophyll) in the origination of protein complexes (e.g., photosynthetic reaction centres): the primary sequences of the subunits of protein complexes do not ‘code’ for the assembly rules.

In summary, in our opinion all the above cases show that phenotypes are not genomically encoded in any straightforward sense. The extra-genomic environment therefore possesses the capacity to causally influence phenogenesis in a way that is not under the control of the genome. Our analysis has hereby focused on molecular phenotypes.^17^ We surmise that when non-molecular phenotypes are taken into consideration, this argument cannot but be reinforced. In fact, the more the causal chain between transcription and phenotypic outcome is long and involving extra-genomic resources, the argument in favour of a genomic interpretation of developmental potential progressively weakens.

### Moving Towards Preformationist Organismal Potentialism

The examples illustrated in Sect. 4 put pressure on preformationist genomic potentialism because they show that the metaphysical thesis that merely the genome is causally ‘responsible’ for or ‘encodes’ the phenotype is biologically indefensible. Any genomically regulated process inevitably recruits extra-genomic resources. This point is valid for any ‘gene product’. As illustrated in Sect. 4.2.2 with the example of a basic and universal developmental process, in order to produce an RNA transcript, RNAPs inevitably rely on NTPs, whose distribution in the cellular environment could be biased, an occurrence in no sense dependent on the genome. What is partially dependent on the genome is the process of NTP synthesis, which is genomically regulated. But, again, NTP biosynthesis ultimately depends on the assimilation from the external environment of precursors (chemical elements and compounds) that are in no sense genomically ‘encoded’. Nevertheless, there is no doubt whatsoever that the processes underpinning genomic change are central to development and, as a consequence, evolution.^18^ Such processes are usually categorised under the umbrella term ‘mutation’, referring to a variety of genomic changes generated by a variety of molecular processes (Koonin 2009). But, as we anticipated in Sect. 2, genomic change (in the terms of the epigenetic landscape representation, the change in the pegs or in their structural organisation) *can only manifest its potential to contribute to phenogenesis within a developing organism embedded in a specific environmental context*. The way in which this process takes place can be conceptualised as “mutation-and-altered-development” (Stoltzfus 2006): it is only within a developing organism embedded in a specific environmental context that, for instance, a point mutation can occur and contribute to the manifestation of a particular phenotype, for instance a developmental novelty such as a novel kind of protein (in the terms of the epigenetic landscape representation, the change in the pegs or in their structural organisation can only produce a change in the pull or direction of the guyropes—and, henceforth, a change in the landscape contour – when embedded in a particular relational context). This process requires transcription, translation, folding etc. and, henceforth, it necessarily involves the causal contribution of extra-genomic resources. Moreover, in Sect. 4.1 we argued that the emergence of developmental novelty is not necessarily dependent on the new genomic capacities produced by genomic change. We argued that the assimilation and functional integration of ubiquitous, and sometimes new, environmental materials should not be assumed, in the absence of empirical evidence, to occur because of previously accumulated genomic change. To assume otherwise is an expression of the geno-centric bias on which preformationist genomic potentialism is founded. Moreover, developing organisms often lose the genomic resources supposedly ‘encoding’ for the regulation of their ontogeny. It is not only the acquisition, but also the loss, of genomic parts that might engender a qualitative developmental change and, eventually, developmental or evolutionary novelty (McShea & Hordijk 2013). For instance, consider the incapacity, due to gene loss, to convert 2-keto-L-gulonolactone to ascorbic acid (i.e., vitamin C) in many primates. Many organisms are also unable to synthesise many of the amino acids they need for protein synthesis, again probably because of gene loss. In such cases, a realistic evolutionary scenario is that these developmental novelties were phenotypically neutral and became fixated in the relative lineage by drift (King & Jukes 1969, p. 792). One of the reasons why it is supposed they were neutral is that organisms can in most circumstances easily compensate the lack of autonomous synthesis through the assimilation of biotic environmental materials. Lack of genomic resources is thus compensated by the assimilation of biotic environmental materials and the simultaneous existence of physiological capacities allowing the developing organism to make use of such materials (West-Eberhard’s “phenotypic accommodation”). Again, the possibility of mutation-and-altered-development requires developmental resources and organismal physiological capacities that seem intuitively additional to genomic ones.

## Debunking Preformationist Organismal Potentialism

Internalist and organicist accounts of developmental potential stress the causal role of those aspects of organismal bodily architecture that are not directly or solely dependent on the genome.^19^ For instance, following St. Hilaire’s (1818, pp. 18–19) tradition, one might identify one strand of the internalist approach as emphasising extra-genomic organismal material composition. For instance, Newman (2021) argues that “aspects of the phenotype are latent in the organism’s material identity and that these features will spontaneously emerge if the conditions are right”. Another strand of internalism stemming from the same tradition emphasises organismal structural organisation. For instance, Austin and Nuño de la Rosa (2021) argue that the possibility of organismal form is determined by the intrinsic dispositions of the developing organism or some of its sub-structures, which exhibit a variety of dispositional properties (e.g., modularity and plasticity), while their developmental potential is variably manifested in response to a variety of environmental conditions. There is no denying that the material composition and structural organisation of developing organisms is of fundamental importance in order to conceptualise developmental potential. At the same time, given that phenogenesis is often co-dependent on a host of additional extra-organismal environmental inputs and materials, unique emphasis on the causal contribution of the developing organism is as unsubstantiated as in the case of preformationist genomic potentialism. Whenever the organismal view downplays the causal role of the extra-organismal environment, it becomes a more encompassing form of an internalist form of preformationism that could be labelled *preformationist organismal potentialism*. We now pass to criticise this view.

Before doing so, a more significant characterisation of the extra-organismal environment is due. As we anticipated in Sect. 2, the extra-organismal environment is constituted by a continuously changing set of abiotic and biotic resources. It includes abiotic physical (e.g., photons) and chemical materials (e.g., carbon dioxide, water) that are necessary for various essential metabolic activities (respectively photosynthesis, nucleotide synthesis and protein folding). The basic chemical dependency of life on the extra-organismal environment is of pivotal importance. It is arguably preposterous to argue that genomically ‘encoded’ enzymes invented life’s biochemistry. Rather, enzymes “merely allow chemical reactions that have a tendency to occur anyway to occur more rapidly” (Martin et al. 2008, p. 811). Indeed, the basic structural features of many metabolic pathways often predate genomic regulation. The original cells probably managed to harness already existing spontaneous and autocatalytic chemical reactions (e.g., the Wood–Ljungdahl metabolic pathway, Martin & Russell 2007) by internalising them in their physiology, continuously fine-tuning them, eventually by regulating them genomically. The abiotic extra-organismal environment might thus be seen as representing the most fundamental causal basis for life’s potential to evolve. This point is so basic that it might be trivialised: no organism can (i.e., has the potential to) survive without entrenching environmentally available resources.^20^ Moreover, some major evolutionary innovations were also made possible by virtue of the assimilation and functional integration of abiotic extra-organismal environmental resources (e.g., iron entrenchment in the heme prosthetic group contributing, in several lineages of animals, to the evolution of protein complexes such as haemoglobins; silicon entrenchment contributing, in some rice lineages, to new forms of fungal infection resistance; stone entrenchment contributing, in several lineages of birds, to gizzard evolution).

The extra-organismal environment also includes a vast array of biotic resources produced by other organisms: the batrachotoxins, vitamin C, amino acids, prosthetic groups such as quinones, as well as DNA resources, nutrients, artificial chemical compounds etc. It finally includes semi-organismal and organismal entities such as viruses, endosymbionts, unicellular organisms etc. that might be entrenched too. The fundamental critical issue is thus whether environmental resources external to the developing organism contribute to increase developmental potential and whether their causal role can be coherently downplayed as the mere ‘triggering’ of the manifestation of the intrinsic dispositions of the developing organism. We shall both argue that they do increase developmental potential and that their causal role is not confined to being ‘triggering’ causes of the intrinsic dispositions of the developing organism.

One critical argument against *preformationist organismal potentialism* concerns the very possibility of identifying the developmental system of reference to which developmental potential should be ascribed. Developing organisms are compositionally and organisationally unstable entities. The characterisation of developmental potential at different developmental stages (e.g., fertilised embryo or adult) might therefore be different. One important aspect of such difference concerns the extra-organismal environmental resources available to the developing organism at different stages. So, given that no developmental stage is the pinnacle of ontogeny (any choice would be arbitrary as development is a continuous process) and given that the environment is constituted by environmental resources that differ at each developmental stage, there is no principled reason to identify which developmental stage is crucial to characterise developmental potential. Hence, characterising developmental potential in terms of the potential of an organism at a particular stage x in isolation from the environment is unwarranted.

Considering a further illustrative example will help elucidating the fundamental limitation of preformationist organismal potentialism. *Elysia chlorotica* is a remarkable slug able to survive several months solely by photosynthesis. Its photosynthetic capacities are due to the developmental entrenchment of the plastids ingested by feeding on the marine algae *Vaucheria*, which are assimilated by phagocytosis and then functionally integrated in the specialised digestive tubular cells in the slug’s digestive epithelium. This evolving symbiotic relationship can be characterised in terms of several developmental and evolutionary novelties at the basis of the physiological integration of the two developmental systems involved. One crucial aspect of this integration concerns the way photosynthetic capacities are manifested. The plastid encodes only one (i.e., RuBisCo) of the 12 essential photosynthetic enzymes (Rumpho et al. 2011, p. 307). The slug acquires via lateral DNA transfer from the plastid the genomic resources from which the enzymes of the photosynthetic pathway are biosynthesised. The enzymes are then exported to the plastid (Rumpho et al. 2008). To be realised, this process requires compatibility between the mechanisms of protein export and import on the parts of the slug and plastids. In fact, the plastids have relinquished two of the four layers of their membrane, facilitating protein transfer (Rumpho et al. 2011, p. 306). Thus, the simplification of the plastid membrane is tailored to the acquisition of the slug-produced proteins.^21^

In this case we have two developmental systems, one more organismal than the other if organismality is characterised in terms of physiological and reproductive autonomy (after all, plastids are endosymbionts). When biotic environmental resources are other developmental systems, it becomes arbitrary to distinguish the developmental unit with respect to which developmental potential is characterised. The same point can be made, by extrapolation, about all “composite” developmental systems (Mahner & Bunge 1997), e.g., all eukaryotes, including all holobionts, such as the multicellular eukaryotes traditionally studied in developmental biology. Conceptually speaking, it is incorrect to consider the organisms of the species *Elysia chlorotica* photosynthetic animals*.* This is because, at the start of ontogeny, they do not possess the capacity to photosynthesise. If the unit of reference is just the slug, it is not a photosynthetic animal. Indeed, the only developmental system that possesses the capacity to photosynthesise is obviously the association.^22^ To retort that the slug possesses the intrinsic disposition to photosynthesise would be equivalent to downplay the causal role of plastids. It would be equivalent to argue that plastids merely ‘trigger’ the manifestation of an intrinsic organismal disposition to benefit from photosynthesis on the part of the slug (in analogy to downplaying the causal role of any environmental input in terms of ‘triggering’ the manifestation of a preformed, latent, intrinsic organismal disposition). Conversely, it might be argued that the slug ‘triggers’ the manifestation of a preformed, latent, intrinsic organismal disposition to import slug-produced proteins on the part of the plastids. The inconsistency of the latter two claims illustrates the fundamental incoherence of preformationist potentialism in general: to focus on the potentials of just one of the interactants of a causal relation is insufficient to explain the very possibility of that causal relation.^23^ In sum, if certain extra-organismal, extrinsic environmental resources can be entrenched by the developing organism in phenogenesis, this is because such resources have the potentiality to contribute to the construction of that very process of development. In brief, the extra-organismal environment represents a distinct causal basis for the organisms’ potential to construct different phenotypes.

## An Interactionist Multi-Causal Basis View of Developmental Potential

In this paper we argue for an interactionist multi-causal basis view of developmental potential construction. According to this view, each of the causal bases (genomic, organismal, environmental) must be seen as necessary but in themselves insufficient sources for development potential. They are *partial* and *complementary* causal bases, only their inter-relation providing the complete causal basis that is needed to ground the complete set of developmental potentialities of an organism. The position defended has two implications: first, developmental potential must be conceived as a genuine extrinsic relational property of organisms; second, developmental potential cannot be seen as the mere actualization of a subset of preformed potentialities given either ab initio or at any other developmental stage.

Let us first clarify our view of developmental potential as a genuine extrinsic relational property. Evoking Love’s analysis of the evolvability disposition and, in particular, the view that “the probabilistic disposition of evolvability instantiated by populations or groups is not wholly definable in terms of intrinsic properties” (2003: 1025), Hüttemann and Kaiser (2018) have argued that there are three main options to deal with any putative example, i.e., *D*, of extrinsic disposition:

(1) *D* may be seen as an *extrinsic* disposition if the external factors to which *D* is typically associated are somehow “over and above mere manifestation conditions”;

(2) On the contrary, *D* should be seen as an *intrinsic* disposition if the external factors to which *D* is typically associated are merely manifestation conditions of *D*;

(3) Finally, *D* may still be seen as an *intrinsic* disposition if *D* is taken to be a collective disposition possessed by the compound system consisting of a given individual and its associated external factors. In this case, intrinsicality is restored, “but at the cost of changing the carrier of the disposition” (2008: 422).

These three views ultimately concern the way in which we conceive the *relationship* between a *disposition* and the *external factors* to which a disposition is typically associated. Let us then consider how the three options fare in the case of developmental potential.^24^

In our view, developmental potential cannot be generally seen as a collective disposition of the compound system constituted by an organism and its external environment, for the very simple fact that only organisms as such are subject to and subjects of development. Given that the only units of development are, in natural conditions (see note 1), organisms, this third option might then only be endorsed when the developing systems constituting the compound system are somehow organismal, as in the case of composite systems like, for instance, *Elysia Chlorotica* (Sect. 5). However, while intrinsicality is restored, somehow incoherently, by changing the carrier of the disposition, the argument we have developed throughout the article concerning developmental entrenchment would challenge even this extension. In fact, even compound systems necessarily rely on environmental inputs and materials. Therefore, we are left with the first two options. In this respect, the fundamental problem, as Hüttemann and Kaiser (2018) rightly note, is whether the external factors to which a disposition is typically associated can be taken as mere ‘manifestation conditions’ for that disposition, or whether they ‘co-determine’ the ‘very nature’ of that disposition, as well as its ‘causal efficacy’ (2018: 422). Therefore, the fundamental question is: are extra-organismic factors “something over and above mere manifestation conditions” of the organism’s developmental potential? The analysis hereby provided supports the view that the external factors which are typically associated to every organism’s potential to develop cannot be seen as mere ‘manifestation conditions’ whenever they are an *integrative part* of the very nature or identity of developmental potential. As we strenuously argued, the developmental potential of an organism includes its potential to causally interact with certain biotic and abiotic environmental materials, to assimilate and, sometimes, functionally integrate them as developmental resources. Particularly when functional integration generates developmental novelty, as in the several examples we considered in Sects. 4 and 5, it must be stressed that the environment is part of the causal basis of the organism’s potential to construct some of its phenotypes. To see this causal contribution just as a manifestation condition is the symptom of an unjustifiable intrinsicalist bias. Consider again the phenomenon of silicon entrenchment as a general exemplification of our position (Sect. 4.1): in this situation, an environmental material becomes an integral part of the organismal phenotype in the sense that without silicon being placed on cells’ walls, the rice plant cannot be resistant to fungal infection. This means that to consider silicon just a ‘trigger’ or ‘manifestation condition’ is a bias because silicon literally *co-participates* in the construction of the novel phenotype. In short, without silicon, the construction of that phenotype would not be a *real possibility*.

As we argued, only the inter-relation of genomic, organismal and environmental factors provides the causal basis that is needed to ground the complete set of developmental potentialities that every organism has. Conceiving the developmental potential of every living organism not as an extrinsic relational property but as an absolutely intrinsic property, can only be explained by the tacit adoption of a one-sided view (centred on organisms or some of its parts) of the possibility itself of the relations between organisms and their actual environments. Again, in an environment without silicon rice plants do not have the potential to assimilate silicon so as to acquire, through its functional integration, the potential of being resistant to fungal infection. To state that rice plants have the potential ‘to functionally integrate *silicon*’ *is* the same as saying that their *relation* is *possible*. Rice plants' potential is thus an extrinsic relational property, whose instantiation implies the *co-existence* of silicon and its instantiation of the *correlative* relational potential of ‘being able to be functionally integrated *by rice plants*’, thereby providing the means for rice plants to acquire another extrinsic relational property, namely, the potential of ‘being able to resist *fungal infection*’. In sum, the developmental potential for many phenotypes may only be possessed by the organisms by virtue of their environments’ properties and available materials. Again, in an environment without silicon, rice plants do not have the power to assimilate and functionally integrate it (so as to acquire the new power of being resistant to fungal infection). For rice plants to acquire that potential as a real power (i.e., for that potential to represent a real physical possibility), they must either change environment or their environment must change. The developmental potential for some phenotypes can thus be acquired and lost by organisms depending on the actual properties of their environments. To reject this view is just to validate what seems to us an unrealistic, abstract notion of real-world potentialities and possibilities. In particular, the mistaken underlying assumption of both the genome- and organism-centric views of developmental potential is to think that, since organisms are the entities that *can*, i.e., have the potential to, develop, the *causal basis* of such potential must be found exclusively in themselves, as self-sufficient and ontically independent entities.

The thesis we support is that developmental powers or dispositions are thus extrinsic relational properties of organisms. This means that the developmental potential of any organism has a combined *endo-exogenous* causal basis. As McKitrick (2014, p. 66) notes:

“the mistake of those who claim that intrinsic duplicates have the same disposition is not that they are only focusing on the causal basis of that disposition, but rather that they are only focusing on *part* of the causal basis of the disposition, and not taking into account the properties extrinsic to the disposed individual that are part of the causal basis of its disposition”.

Furthermore, as we have argued, the developing organism’s potential to entrench certain resources from its environment (a necessary type of developmental interaction, see Sects. 4.3 and 5) implies the *co*-*existence* of correlative potential of such resources to be entrenched by the organism. In fact, the organism *cannot* (i.e., it does *not have* the potential to) entrench from the environment *everything* or *whatever*. Entrenchment is possible given the material composition and structural organisation of the developing organism. However, the real potential for entrenchment also depends on the existence of specific environmental structures with their associated sets of correlative potentials to be entrenched. *Some* environmental resources *can* (i.e., have the potential to) be used by the developing organism as contributing causal factors for development, while others *cannot* (i.e., they lack that correlative potential). So, the very nature, identity or definition of developmental potential integrates:

–specific types of causal *relations* (viz., assimilation and functional integration), and.

–specific types of causal *co-relata* (viz., certain biotic and abiotic environmental materials).

This means that the individuation of the developmental potential for many phenotypes exhibits the defining feature of what is considered to be an *extrinsic relational property*, that is, the fact that the individuation of a property necessarily involves a reference to *specific relations* between the bearer of that property and some *external* entities as its co-relata. In biological practice, it might make perfect sense to discriminate cases in which the environment merely ‘triggers’ phenogenesis instead of being a ‘constitutive’ element of the phenotype (this is why we have distinguished, following West-Eberhard, environmental inputs and materials, see Sect. 2). What we object to is the metaphysical interpretation of biological phenomena. As we diagnose the problem, internalists (of any kind) view developmental processes from a one-sided perspective instead of relationally, i.e., by focusing on the intrinsic properties of developmental systems instead of considering the environment as extending the developmental system’s developmental potential. What we argue is that this is a symptom of an unjustifiable metaphysical bias that is difficult to justify, especially when there exists a coherent relational alternative. And this explains why we focus our narrative on cases of developmental entrenchment of materials rather than inputs: the functional integration of environmental materials is hardly interpretable as a process of mere triggering of latent intrinsic potential.

Finally, we argue that, given that the relational construction process of development takes time and that developmental potential never becomes fixed at a particular developmental stage, it cannot but be epigenetic: “developmental potentialities change as development proceeds” (West-Eberhard 2003, p. 13). The developmental potential of any developing organism does not solely depend on the genome it possesses or the particular causal capacities endogenous to the developing organism that is capable of manifesting at a specific developmental stage. Development is a never-ending process, a process that starts at fertilisation and ends with death; so, for instance, to consider the ‘adult-stage’ as the finalisation of the process is meaningless.^25^ It becomes therefore preposterous to consider any developmental stage as the pinnacle of ontogeny and to consider the causal capacities acquired at that specific stage the maximisation of developmental potential on the basis of which a preformationist narrative is then superimposed. Rather, developmental potential is constructed by the developing organism through ontogeny by interaction with the environment, that is, through the assimilation and functional integration of abiotic and biotic extra-genomic and extra-organismal developmental resources such as chemical compounds, nutrients, DNA molecules, symbionts, etc.

Since both the *intra*-organismal environment and the *extra*-organismal environment undergo *structural changes*, thereby instantiating *new structural properties*, they can also acquire different *potentialities* in the course of time. Therefore, many changes in the *intra*-organismal or *extra*-organismal environments may bring about the acquisition of novel developmental capacities at any ontogenetic stage. Environmental interaction endows developing organisms with causal capacities that might be developmentally (and henceforth evolutionarily) novel and that in no significant biological sense are latent and dormant either in the genome or the developing organism taken at a particular developmental stage. As we tried to show, novelty often requires environmental construction through the assimilation and functional integration of environmental materials in phenogenesis. Through development, the environment does not merely activate or trigger the manifestation of fixed, pre-set and preformed potentialities. It rather generates new ones, most obviously new with respect to the lineage to which the organism belongs. The necessity of environmental contextualisation makes environmental inputs and materials often necessary not only to actualise some already existing potentialities, but also to create novel developmental dispositions. This means that the developing organism can acquire novel developmental potentialities that were not possessed by or present in its previous structural states. The set of developmental potentialities available to every organism thus changes during ontogeny and, in this sense, we refute any form of preformationist potentialism. In brief, the potency of a developing organism is malleable through *environmental engineering*.^26^ The unpalatable alternative is to consider the qualitative developmental changes at the basis of evolutionary novelty mere actualisations of a prefixed set of fundamentally intrinsic potentialities either given ab initio in the genomic or organismic structure or acquired at later developmental stages by the developing organism independently of the extra-organismic environment. The former view implies that, ultimately, the developmental potential of all living organisms would be somehow metaphysically derivable from the original potentialities of the primitive cell’s structure. The latter view avoids this absurd limitation, nevertheless maintaining an intrinsicalist bias. Both preformationist genomic potentialism and preformationist organismal potentialism capture a partial truth. But this partial truth hides an important fallacy: neither the genome nor the developing organism possess, in a latent form, all developmental potentialities. Developmental potential is not meaningfully ascribable to biological entities when considered as isolated and abstracted structures. Indeed, all the examples illustrated so far referred to environmental resources such as the inputs by means of which developing organisms regulate phenogenesis (e.g., the error-proneness of RNAPs, the biased distribution of NTPs, the presence of certain abiotic and biotic chemical compounds) and the abiotic and biotic materials that developing organisms deploy for phenotype construction (e.g., silicon, batrachotoxins, stones, amino acids, prosthetic groups, plastids). Often environmental contribution involves biotic materials’ entrenchment (with, at the extreme, the entrenchment of complex semi-organismal—e.g., plastids, see Sect. 5—or organismal developmental systems).

On this basis, we can refute not only any mono-causal basis view of developmental potential, but also any intrinsicness-based form of preformationist potentialism. We also argue that the only biologically and metaphysically coherent conceptualisation of development is relational and epigenetic. The main advantage of the proposed characterisation of developmental potential is that it bridges biology and philosophy, specifically the findings stemming from ecological developmental biology and the endo-exogenous causal basis view of developmental potential as a genuine extrinsic relational property of the organisms.

## Footnotes

1 Even though also the cells and organs of multicellular organisms might develop in controlled experimental conditions, we will not consider them as units of development because they are organismal parts.

2 Throughout this paper, we shall use the term ‘extrinsic relational property’ in order to contrast it with the so-called class of ‘intrinsic relational properties’. An intrinsic relational property of an entity may either be a relational property between some of its *proper parts* (e.g., Peter has the property of having longer legs than his arms), or a *self-reflexive* property (e.g., the property of Peter of being identical to *itself*). In this sense, to be a relational property does not necessarily mean to be an extrinsic property. See Marshall and Weatherson (2018, Sect. 2.1).

3 This position is compatible with the possibility that, in some cases, some dispositions might be acquired by developing organisms merely because of their intrinsic properties (e.g., “spontaneous”—i.e., not environmentally-induced -, mutations or changes in the topological properties of a gene network). What we shall deny is that any mono-causal view is sufficient to capture all possible cases of acquisition of developmental potentialities.

4 Even though Love focuses on a dispositional property of populations or lineages (i.e., evolvability or evolutionary potential) while we focus on a dispositional property of developing organisms (i.e., “developability” or developmental potential), our analyses share an externalist ethos.

5 This assumption becomes contentious if the genome is seen, for instance, as a “read-write” memory system edited through natural genetic engineering processes (Shapiro 2011) rather than as a “read-only” memory system or static repository of information.

6 A chemical compound (e.g., a nucleoside triphosphate) harnessed for phenotype construction by a cell (e.g., to produce an RNA molecule) is a *material*. The biased concentration of chemical compounds might be considered an *input* if it causes a particular kind of behaviour on the part of a molecular entity (e.g., a protein) that would not otherwise occur.

7 We use the term ‘qualitative’ in order to refer to changes in composition, organisation or function of the responsive biological system.

8 The idea can be articulated simplistically as follows: an organism with only two genotypes—correlated bijectively to the only two phenotypes it can manifest in different variants—will have a genome that limits the organism’s developmental potential within the upper and lower boundaries of the genotypes’ reaction ranges.

9 The focus of our analysis in this section is on the extra-organismal environment but, by analogy, the upshot is the same in the case of the extra-genomic environment internal to the developing organism.

10 Being uniquely causally responsible, the genome is also causally primary. The debate concerning causal parity is peripheral in this context.

11 The novelty of the environment is better understood in an evolutionary sense, for instance as the presence of a new environmental material never encountered by any organism of the lineage. The reason for this characterisation is that many more environments might be new for the individual organism than for the lineage. In this way, we partially pre-empt the counterargument that the genome of the organism has already acquired, through evolution, the genomic capacities to developmentally entrench the new environmental material.

12 Developmental novelty might be saltational and abrupt. For instance, changes undergone by proteins triggered by the developmental entrenchment of chemical precursors such as chemical elements (e.g., iron in heme groups) and chemical compounds (e.g., quinones in photosystem II) might have produced new phenotypes (i.e., haemoglobins and photosynthetic reaction centres) abruptly in the absence of genomic change. We articulate this point in Sect. 4.2.2.

13 Our argument concerns the origin of developmental novelty, not how it can be inherited, which is of course a fundamental biological issue.

14 Note that, when examined from the point of view of their potential evolutionary novelty, the above examples vary in significance. The functional integration of silicon has not generated a new species of plant. It is an embellishment in this sense. When we move to the integration of batrachotoxins, however, we see that new organismal types (i.e., a new species such as *Phyllobates terribilis* and, arguably, the new genus *Phyllobates* and family Dendrobatidae) have evolved, even though not a new phylum or a new body plan.

15 The RNAP is ‘coded’ by the genome and, henceforth, it might be argued that the latter is causally ‘responsible’ for the former’s structure. This argument is fallacious for the general reason that phenotype construction necessitates the recruitment of extra-genomic developmental resources in no sense encoded by the genome (see Sect. 4.3).

16 Of course, an explanation must be provided for how a developmental novelty can be inherited, at least initially, non-genetically.

17 Protein folding is one of the most basic, phylogenetically universal, developmental processes. Given that it is frequently described in terms that are compatible with preformationist genomic potentialism (e.g., genes “coding” for proteins), we find it important to show how the functional integration of environmental resources affects protein folding.

18 Waddington’s genetic assimilation (Sect. 3.3) captures an aspect of this causal role of the genome. Natural genetic engineering (see note 5) another.

19 The preformationist genomic potentialist might rebut that all bodily resources are ultimately genomically ‘encoded’. This is clearly not true, as, for instance, cell membranes and mitochondria show. Furthermore, the cells of distant lineages can appropriately interact despite substantial genomic divergence. For instance, embryonic stem cells of the teleost fish medaka (species *Oryzias latipes)* and blastomeres of zebrafish (species *Danio rerio*) form viable chimeric organisms despite the two species diverging around 320 million years ago (Hong et al. 2012). Presumably, the cells of these organisms manage to appropriately interact because they share both genomic *and* non-genomic developmental resources.

20 Internalist approaches often stress the fundamental importance of thermodynamic openness. At the same time, they also frequently emphasise properties of developmental systems ranging from self-production, organizational invariance and closure. If such emphasis is aimed to downplay the causal role of the extra-organismic environment in extending developmental potential, it is a symptom of an intrinsicalist bias.

21 In no way the transfers of DNA and proteins between developmental systems supports preformationist genomic potentialism. This is because any developmental resource acquired from another developmental system is extrinsic, i.e., extra-organismic.

22 A similar point is made by Nuño de la Rosa et al. (2021).

23 It must be stressed, as already indicated in note 3, that we do not deny that some dispositions might be acquired by developing organisms merely because of their intrinsic properties. What we deny is the scope of intrinsicalism as expounded in preformationist genomic and organismal potentialism alike. Needless to say, functional integration cannot happen in a vacuum: it needs organismal capacities. What we also argue – and makes our position different from any kind of preformationist potentialism—is that the organismal capacities for achieving functional integration are in no coherent sense preformed or latent.

24 A similar contrast between extrinsic and intrinsic pertains to other debates on the nature of biological dispositions (see Hütteman and Kaiser 2018 and Brigandt et al. 2023).

25 See Nuño de la Rosa (2010) for a contextualization and Minelli (2003) for a critique of this position.

26 It might be argued that our illustrative examples do not distinguish developmental processes that define the type from those that embellish developmental systems and that, therefore, we do not show that the developmental novelty caused by the functional integration of environmental materials can produce significant evolutionary novelties leading to lineage differentiation. It seems to us arbitrary to dismiss the evolutionary effects of developmental entrenchment as mere embellishments. Indeed, the functional integration of environmental materials (e.g., silicon, iron in hemes, batrachotoxins, stones, hemes in haemoglobin, plastids) engenders novelty with different kinds of evolutionary effects, some of them clearly significant. Stretching the criticism, we would have to dismiss, for example, the original eukaryote as a mere embellishment. As West-Eberhard (2003, p. 500) has in our opinion correctly noticed with characteristic insight: “*Entrenchment of some environmental elements is so thorough and widespread that we forget they were once evolutionary innovations*”. We take her point biologically seriously and have tried to unravel its philosophical implications throughout the article.
